# Appropriateness and Abuse of Antipyretics, Anti-Inflammatory Drugs and Antibiotics in Children and Adults

**DOI:** 10.3390/antibiotics15050436

**Published:** 2026-04-27

**Authors:** Giangiacomo Nicolini, Massimo Crapis, Andrea Lo Vecchio, Roberto Parrella

**Affiliations:** 1 Pediatric Unit, Ospedale di Conegliano (TV), 31015 Conegliano, Italy; 2 Infectious Diseases Unit, Azienda Ospedaliero Universitaria di Ferrara, Ospedale di Cona, 44124 Ferrara, Italy; massimo.crapis@ospfe.it; 3 Department of Translational Medical Sciences, University of Naples Federico II, 80138 Naples, Italy; andrea.lovecchio@unina.it; 4 Respiratory Infectious Diseases Unit, Azienda Ospedaliera Specialistica dei Colli, Cotugno Hospital, 80138 Naples, Italy; roberto.parrella@ospedalideicolli.it

**Keywords:** antipyretics, anti-inflammatory drugs, antibiotics, paracetamol, ibuprofen, antimicrobial resistance, stewardship, pediatrics, adults, infectious diseases

## Abstract

Anti-inflammatory agents, antipyretics, and antibiotics are commonly used to manage fever and pain associated with infectious diseases in both adults and children. Despite their effectiveness, inappropriate and unnecessary prescriptions remain widespread, leading to adverse patient outcomes and, in the case of antibiotics, contributing to antimicrobial resistance. Addressing these issues requires effective stewardship programs focused on educating healthcare professionals and the public on evidence-based guidelines for optimal prescribing practices. This paper explores the five “A”s fundamental to infection management in pediatric and adult patients: appropriateness, abuse, antipyretics, anti-inflammatory agents, and antibiotics. Through a comprehensive literature review, expert perspectives, and clinical guidelines, the study evaluates the roles of anti-inflammatory agents (e.g., ibuprofen), antipyretics (e.g., paracetamol), and antibiotics in clinical practice, highlighting best practices for their use. Current guidelines emphasize that antipyretics should only be administered when fever is accompanied by significant discomfort or pain, as fever itself plays a role in the immune response. Based on the available literature, experts also suggest that paracetamol should be preferred as a first-line antipyretic due to its favorable safety profile, while ibuprofen should be used with caution, particularly during respiratory infections, varicella, and severe bacterial infections, due to its potential to exacerbate complications. According to experts, special consideration is also required for patients with renal or gastrointestinal comorbidities to prevent toxicity. Regarding antibiotics, prescriptions should be limited to clear evidence of bacterial infection to avoid unnecessary patient exposure and the development of antimicrobial resistance. Stewardship programs underscore the importance of selecting the right agent, optimizing dosing, and introducing shorter treatment regimens where supported by evidence, to improve therapeutic outcomes while minimizing resistance risks. Ultimately, this paper provides practical, evidence-based recommendations to support rational prescribing of antipyretics, anti-inflammatory drugs, and antibiotics, aiming to optimize patient outcomes, prevent unnecessary toxicity, and contribute to global efforts against antimicrobial resistance.

## 1. Introduction

Fever and pain are among the most common reasons for primary care consultations and hospital admissions, often presenting as challenging symptoms in children and adolescents [1,2]. They are often associated with various infectious diseases, including upper and lower respiratory tract infections (RTIs, i.e., flu-like syndrome, pneumonia), and urinary tract infections (UTIs). Fever, in particular, plays a critical role in the immune response against infectious agents, as it contributes to inhibiting pathogen replication and enhancing the activity of immune cells such as neutrophils, macrophages, and lymphocytes [3].

Non-steroidal anti-inflammatory drugs (NSAIDs) and antipyretics are widely used to manage fever and pain in both pediatric and adult populations, having demonstrated both high efficacy and a favorable safety profile in various clinical settings [4]. However, their use in clinical practice often deviates from evidence-based recommendations, leading to cases of misuse and abuse [5,6]. Specifically, the management of fever is characterized by overtreatment, often owing to “fever phobia” [5], whereas pain is often undertreated, leading to untimely and inadequate analgesia [6].

The inappropriate use of NSAIDs has been associated with important adverse effects, such as gastrointestinal bleeding, kidney damage, cardiovascular complications, and partial immune suppression [7]. Moreover, NSAIDs may mask the symptoms of bacterial infections, potentially delaying diagnosis and increasing the risk of superinfections [8,9]. On the other hand, overuse of paracetamol is recognized as a potential risk factor for hepatotoxicity [10].

Antibiotics also represent a critical component of the pharmacological management of infectious diseases in both hospital and community settings [11]. Despite their widespread use, evidence suggests that 20–50% of antibiotic prescriptions in children are either unnecessary or inappropriate. This represents a significant public health challenge, increasing the risk of adverse drug reactions, elevating healthcare costs, and contributing to the growing problem of antimicrobial resistance (AMR) [12]. In response to this challenge, antibiotic stewardship programs have become essential. These initiatives aim to address inappropriate prescribing practices by educating healthcare professionals and the public, encouraging adherence to evidence-based guidelines, emphasizing optimal drug selection, and dosing, and fostering professional networking and peer support among physicians [11,12,13,14].

In the context of improving clinicians’ education on rational drug prescription, this paper aims to explore the five “A”s involved in managing infections in children and adults: appropriateness, abuse, antipyretics, anti-inflammatory agents and antibiotics. By reviewing literature-based evidence, incorporating experts’ perspectives and drawing insights from adult and pediatric guidelines, the study evaluates the role of anti-inflammatory agents (e.g., ibuprofen) and antipyretics (e.g., paracetamol) as first-line treatments for managing fever in patients with infectious diseases. Moreover, the study summarizes optimal antibiotic prescribing strategies across various clinical scenarios, providing comprehensive tables that may serve as practical tools to clinicians in daily practice. These suggestions aim to improve clinical decision-making, optimize patient outcomes, and contribute to the global effort against antimicrobial resistance.

## 2. Methods

This manuscript was developed as a narrative, evidence-informed review supported by the clinical experience of specialists from the Italian Society of Pediatric Infectious Diseases (SITIP) and the Italian Society of Infectious Diseases (SIMIT). The contributing experts were clinicians with long-standing experience in pediatric or adult infectious diseases.

To prepare the document, each expert reviewed the available scientific literature relevant to their area of contribution. Evidence was identified through searches of major biomedical databases, including PubMed/MEDLINE, EMBASE, Scopus, and the Cochrane Library, covering publications approximately from 2000 to April 2025. The search strategy combined free-text terms and controlled vocabulary (MeSH/Emtree), adapted for each database. Core search terms included: (“appropriateness” OR “abuse”) AND (“paracetamol” OR “acetaminophen”) AND (“ibuprofen” OR “NSAIDs” OR “non-steroidal anti-inflammatory drugs”) AND (“antibiotics” OR “antimicrobial agents”) AND (“antimicrobial stewardship” OR “antimicrobial resistance”) AND (“fever” OR “pain management”) AND (“drug safety” OR “adverse effects”). These terms were combined using Boolean operators (AND/OR), and filters were applied when appropriate to restrict results to human studies. National and international guidelines and relevant regulatory safety communications (e.g., European Medicines Agency (EMA) and Italian Medicines Agency (AIFA)) were also examined.

Studies were eligible for inclusion if they met the following criteria: (i) written in English; (ii) addressed the clinical use, pharmacological profile, safety, efficacy, appropriateness or abuse of antipyretics, anti-inflammatory medications or antibiotics; (iii) involved pediatric and/or adult populations. Case reports, small case series, and preclinical or in vitro studies without clear clinical applicability were excluded.

The screening and selection of studies were performed by the contributing authors based on relevance, methodological quality, and consistency with the objectives of the review. Preference was given to randomized trials, systematic reviews, meta-analyses, large observational studies, and official guideline documents. Given the narrative nature of the manuscript, a formal systematic review process (e.g., Preferred Reporting Items for Systematic Reviews and Meta-Analyses flow diagram) was not applied.

The draft manuscript underwent several rounds of informal discussion among the contributing authors. Content was refined through iterative feedback, with disagreements addressed through direct discussion until a shared interpretation was reached. This process reflects expert collaboration rather than a structured consensus method, and the resulting recommendations should be interpreted as expert opinion informed by available evidence, not as guideline-level statements.

## 3. The 5 As: Appropriateness and Abuse of Antipyretic, Anti-Inflammatory Drugs and Antibiotics

### 3.1. Appropriateness and Abuse

Appropriateness and abuse represent two interrelated dimensions of pharmacological management, which are highly relevant in the context of infectious diseases. Appropriateness is a multidimensional concept encompassing clinical and pharmacological considerations, including timely initiation of therapy, appropriate drug selection, dosing, route of administration, duration, and adherence to clinical guidelines [15]. In contrast, abuse or misuse refers to deviations from these standards, including inappropriate or unnecessary use of medications that do not align with evidence-based indications [16].

These two concepts are explored in the following sections in relation to antimicrobial stewardship and anti-inflammatory and antipyretic prescribing. Antimicrobial stewardship plays a key role in promoting appropriate antibiotic use, improving clinical outcomes, and minimizing toxicity and antimicrobial resistance [15,16]. The same principles apply to anti-inflammatory and antipyretic therapies, where prescribing decisions should be guided by clinical indications, therapeutic goals, and safety considerations [17].

### 3.2. Antipyretics and Anti-Inflammatory Drugs

#### 3.2.1. Overview

Paracetamol and ibuprofen are the two most widely used medications for the management of fever and pain in both pediatric and adult populations. In children, they are the only antipyretics recommended by international guidelines, with paracetamol approved from birth and ibuprofen from the age of three months [18,19]. While no substantial differences have been observed in their overall efficacy, their safety profiles vary depending on the patient’s underlying health status and clinical context, underscoring the importance of selecting the most appropriate agent on a case-by-case basis [18,19,20].

According to current guidelines, antipyretics should be prescribed only when fever is associated with evident discomfort or pain, which in children can manifest as crying, irritability, decreased activity, appetite loss or sleeping difficulties [19,20,21]. Guidelines and supporting evidence also highlight that inappropriate or excessive use, particularly in pediatric populations, can increase the risk of adverse effects and toxicity [17,18,21].

Although pediatricians show a reasonable awareness of recommended antipyretic and anti-inflammatory use, non-evidence-based practices still persist, including the alternating use of paracetamol and ibuprofen, and their prophylactic administration prior to vaccinations [1,22,23]. A persistent tendency toward “fever phobia” among healthcare professionals contributes to the routine, and often unwarranted, prescription of antipyretics, despite current evidence recommending selective administration guided by patients’ underlying comorbidities and clinical presentation [24]. In line with these prescribing patterns, paracetamol remains the preferred option for fever treatment in the emergency department setting as reported in an Italian single-center observational study [25]. Among adults, misuse and abuse are further exacerbated by self-medication practices, a phenomenon that has been exacerbated during the COVID-19 pandemic [26]. Real-world evidence from Italy, derived from data predominantly collected in adult populations, indicates increasing NSAID use from 2019 to 2022, with frequent prescriptions at higher doses and in patient groups not fully aligned with approved or reimbursed indications, raising further concerns regarding appropriateness in routine clinical practice [27].

#### 3.2.2. Use During Infectious Diseases

##### Respiratory Infections

As shown by a recent systematic review, the use of antipyretics does not significantly alter the duration of illness in upper and lower RTIs [28]. While these agents may provide symptomatic relief, their use should be carefully considered when fever is well tolerated, given their potential side effects.

Some evidence also suggests that NSAIDs should be used with caution during active RTIs. A UK cohort study reported that exposure to NSAIDs may be associated with poorer outcomes, including increased risks of hospital admission, in patients with acute RTIs or UTIs [29]. In addition, several retrospective observational studies have reported a significant increase in the risk of complications, such as peritonsillar abscess, in children treated with ibuprofen for acute sinusitis or pharyngitis [30,31,32]. Other observational data in patients with pneumonia have similarly reported an association between NSAID exposure and severe complications such as pleural empyema, pulmonary cavitations and abscesses [33,34,35,36,37,38]. Notably, although the role of NSAIDs in the pathogenesis of complicated bacterial infections is still unclear, it has been hypothesized that their anti-inflammatory activity may affect neutrophil chemotaxis and activity, thus reducing local antibacterial activity and increasing the risk of bacterial complications, particularly if NSAIDs are administered without concomitant antibiotic therapy [37].

##### Varicella

Evidence from national UK guidance sources advise against the use of NSAIDs in patients with active varicella, due to the increased risk of secondary bacterial infections [39]. A multicenter prospective study involving pediatric inpatients with varicella has in fact shown a higher incidence of secondary skin and soft tissue infections among those treated with NSAIDs [40].

##### Serious Bacterial Infections

NSAID use in the context of serious bacterial infections has also raised potential concerns. A UK population-based surveillance study found that patients receiving NSAIDs had a three-fold increased risk of developing streptococcal toxic shock syndrome in the setting of severe *Streptococcus pyogenes* infection [41]. Similar findings were reported in a systematic review and meta-analysis, which concluded that non-selective NSAIDs may mask early signs and symptoms of Group A *Streptococcus* infection, thus delaying antibiotic initiation and increasing the likelihood of severe sepsis, shock and mortality [42].

In 2024 a French pharmacovigilance report documented 216 cases of serious bacterial infections associated with NSAIDs use (162 with ibuprofen, 54 with ketoprofen) over a 4.5-year period. Streptococcal infections were most common with ibuprofen (62%) compared with ketoprofen (44%), they presented as invasive infections in 97% of the cases and included serious manifestations such as severe sepsis or toxic shock syndrome, pleuropneumopathy, meningitis or meningoencephalitis, and necrotizing dermohypodermatitis [43].

##### COVID-19

Evidence suggests that during the COVID-19 pandemic the inappropriate use of antipyretics and anti-inflammatory drugs has increased, often prompted solely by a positive SARS-CoV-2 test result [44]. The Italian Society of Pediatric Infectious Diseases recommends the use of paracetamol (10–15 mg/kg every 4–6 h) in cases of fever exceeding 38.5 °C in children with mild, moderate, severe, or critical COVID-19 infection [45]. Based on these recommendations, and on the evidence of potentially increased safety risks following ibuprofen administration, as detailed in the next paragraph [46,47,48], experts also suggest that ibuprofen should be avoided in children with COVID-19 infection who exhibit vomiting, diarrhea, or dehydration, due to the increased risk of kidney injury. Although some authors initially hypothesized a possible link between ibuprofen use and a more severe course of SARS-CoV-2 infection [49], subsequent evidence refuted this claim. Consequently, the European Medicines Agency and other institutions did not contraindicate the use of NSAIDs in patients with COVID-19 [45].

#### 3.2.3. Safety Considerations

Both ibuprofen and paracetamol are generally considered safe when administered at appropriate therapeutic doses; however, differences in the incidence of adverse effects between the two drugs should be carefully evaluated considering patients’ age and underlying condition [50,51,52,53], comorbidities and concomitant medications [19].

##### Pediatric Population

- Renal safety

One of the primary concerns with both drugs is their impact on renal function, as they reduce prostaglandin synthesis, which plays a critical role in maintaining renal blood flow. Although both drugs can alter renal hemodynamics, paracetamol mainly exerts its inhibitory action on the synthesis of prostaglandins at a central level, in contrast, ibuprofen has either central or peripheral activity and hence shows a more pronounced effect on glomerular filtration rate compared to paracetamol [54,55].

For that reason, the risk of acute kidney injury should therefore be carefully assessed in children when prescribing ibuprofen even at standard therapeutic doses [46,47,48]. Notably, some authors, based on observational findings and literature reviews, suggest that ibuprofen should be avoided in children receiving Angiotensin-Converting Enzyme (ACE) inhibitors or diuretics [36] and that it should be used with caution in children experiencing dehydration [56] or when co-administered with nephrotoxic drugs, including certain antibiotics [38,57].

Antibiotics themselves can contribute to renal toxicity, particularly through immune-mediated mechanisms. Several classes—including penicillins, cephalosporins, macrolides, rifampicin, glycopeptides (i.e vancomycin), and tetracyclines—have been implicated in renal adverse effects in children [38]. Specifically, amoxicillin and cephalosporins warrant particular attention as they carry a risk of nephrotoxicity Given their frequent use as well as their known potential for interaction with NSAIDs, some authors suggest that careful consideration is warranted when these agents are co-administered with ibuprofen [58].

- Gastrointestinal safety

Additional concerns associated with NSAIDs use include gastrointestinal bleeding and complications, which some evidence suggests may be more likely when NSAIDs are co-administered with corticosteroids, such as dexamethasone, beclomethasone, betamethasone, hydrocortisone and prednisolone [38].

- Hepatic safety

In contrast, based on literature evidence experts believe that paracetamol may be regarded as the option with a more favorable safety profile. Nevertheless, according to a systematic review and a case-series, its use should be carefully monitored in children with liver dysfunction, as acute overdose can lead to hepatotoxicity [59,60]. Caution is also advised, based on available literature evidence, when paracetamol is prescribed to malnourished or obese children, or to those receiving long-term treatments with carbamazepine, isoniazid, phenobarbital or other barbiturates [57,60,61].

##### Adult Population

In adult populations, ibuprofen is generally well tolerated; however, real-world pharmacovigilance data indicate that adverse events are relatively common and predominantly involve the gastrointestinal, nervous, and cutaneous systems. Gastrointestinal disorders represent the most frequently reported adverse events in adults, including nausea, vomiting, dyspepsia, abdominal pain, and diarrhoea, reflecting the known effects of cyclooxygenase inhibition on gastric mucosal protection. Additional reported adverse events include dizziness, headache, tinnitus, and visual impairment, suggesting involvement of the central nervous system. Compared with pediatric populations, adults appear to have a higher frequency of gastrointestinal adverse effects and may more frequently report subjective symptoms [62]. Inappropriate prescribing patterns, such as the use of high doses and prolonged treatment durations, may further increase the risk of adverse outcomes, including gastrointestinal complications (e.g., ulcers, bleeding, and perforation), as well as cardiovascular and renal events [27].

Regarding antibiotics, amoxicillin warrants particular caution, as increasing evidence suggests that its nephrotoxic potential may be underestimated. Documented complications include acute interstitial nephritis and crystal nephropathy, both of which may lead to acute kidney injury, particularly in the presence of predisposing factors such as high doses or renal impairment [58]. Similarly, third-generation cephalosporins have been associated with renal adverse effects, including nephrolithiasis, immune-mediated hemolytic anemia, and acute interstitial nephritis [58].

#### 3.2.4. Monotherapy vs. Combinational Therapy

While a multimodal combination of paracetamol and ibuprofen can be beneficial for pain management due to their synergistic effects, their combined use is not recommended for fever treatment according to Italian and National Institute for Health and Care Excellence (NICE) guidelines [18,19]. A systematic review suggests that, while combining or alternating these drugs can reduce fever more effectively than using a single agent, it does not offer a clinically significant benefit to the overall well-being of patients [63]. These findings align with most international guidelines, which recommend monotherapy over combined or alternated treatment [18,64].

#### 3.2.5. Prophylactic Use During Vaccination

The routine use of paracetamol or ibuprofen for the prophylaxis of fever or pain in children undergoing vaccination is not recommended by Italian guidelines [18]. This recommendation is partially based on evidence suggesting that antipyretic use may interfere with the vaccine-induced immune response. Post-vaccination fever plays in fact a key immunological role, promoting the production, proliferation, and migration of neutrophils and T lymphocytes, enhancing both innate and adaptive immune responses and improving vaccine-induced immunity [65,66,67].

Clinical studies have reported a reduction in antibody response in children who received prophylactic paracetamol or ibuprofen at the time of vaccination [68,69]. Specifically, paracetamol has been associated with diminished antibody responses to pneumococcal antigens, while ibuprofen may attenuate responses to pertussis and tetanus antigens [70]. Although no difference in the antibody titer response was observed after receiving a buster dose of pneumococcal conjugate vaccination [69], in 2015 the World Health Organization position paper renewed its recommendation against “the use of analgesics (paracetamol and ibuprofen) before or at the time of vaccination” [71].

#### 3.2.6. Clinical Implications

Effective management of fever and pain ultimately requires tailoring the choice of antipyretics and analgesics to the patient’s specific clinical profile. As suggested by international guidelines [18,21], antipyretics should be administered to alleviate discomfort and pain, rather than solely to reduce body temperature, as fever may serve as a valuable marker of disease progression and patient’s response to treatment. Factors such as liver and kidney function, concomitant medications, and the patient’s overall clinical condition should also be considered to maximize symptom relief while minimizing the risk of side effects [17].

While both paracetamol and ibuprofen are recommended as first-line antipyretics, experts generally view paracetamol as the preferred option in both pediatric and adult patients presenting with fever > 38 °C or discomfort. In children, the recommended dosage is 10 mg/kg for those weighing <5 kg, and 15 mg/kg for those weighing >5 kg (Table 1); while in adults, the maximum daily dose should not exceed 3 g (Table 2). Based on literature evidence, experts also advise that ibuprofen (recommended dosage 10 mg/kg every 6–8 h, maximum daily dose 30 mg/kg) should be used with caution, particularly in the context of RTIs, where its use has been associated with an increased risk of complications [29,33,34,35,36,37,38,39,40], in UTIs, due to safety concerns [29], and in acute intestinal infections/acute gastroenteritis due to the risk of dehydration associated with secretory diarrhea in pediatric patients and the heightened risk of intestinal bleeding [38].

### 3.3. Antibiotics

#### 3.3.1. Antibiotic Prescribing Patterns in Italy

In Italy antibiotics are the most commonly prescribed drugs, particularly in the outpatient setting and among children, due to the high incidence of infectious diseases in this population [78]. Concerns regarding inappropriate prescribing practices have been repeatedly raised, as highlighted in two point-prevalence surveys conducted over the past decades and further confirmed by the latest report from the Italian Medicines Agency [11,78,79]

A point-prevalent survey conducted in 2012 in seven Italian institutions found that 38.9% of hospitalized children had an on-going prescription for one or more antibiotics [11]. Among neonates (<30 days old), 62.8% of prescriptions were for prophylaxis, predominantly for perinatal conditions, whereas in children (≥30 days to <18 years), 64.4% were for active infections, particularly lower RTIs (LRTIs) and febrile neutropenia in oncology patients. The most frequently prescribed antibiotics were penicillins and aminoglycosides in neonates, whereas third-generation cephalosporins and penicillin–beta-lactamase inhibitor combinations were more commonly used in older children. Notably, 60.9% of patients received combination therapy. Several prescribing patterns were deemed inappropriate, including the excessive use of third-generation cephalosporins (e.g., ceftriaxone) for LRTIs and surgical prophylaxis in children, a disproportionately high prescription rate of carbapenems (8.9%) compared to other European countries (4.2%), and the widespread use of quinolones despite their restricted indications [11].

A follow-up study conducted in 2018 indicated a 4% reduction in the annual antibiotic prescription rate per child between 2012 and 2018. However, broad-spectrum antibiotics, particularly third-generation cephalosporins and co-amoxiclav, continued to be frequently prescribed, and preschool-aged children remained highly exposed, with an average of more than one antibiotic prescription per year [79].

The 2022 report by the Italian Medicines Agency further confirmed the persistence of high antibiotic prescription rates and inappropriate prescribing patterns. The report revealed that three out of ten citizens received an antibiotic prescription, with an overall consumption of 21.1 defined daily dose/1000 inhabitants per day, representing a 23.9% increase compared to 2021 and reaching similar levels to those registered before the COVID-19 pandemic. Notably, higher antibiotic use was observed in the extreme age groups, with the highest prevalence in children under four years of age (45.4% in males and 42.9% in females) and in adults aged 85 years or older (59.2% in males and 53.8% in females). The report also highlighted that approximately one-third of children (≤13 years) received at least one systemic antibiotic prescription during the year, with a notable increase compared to 2021. Exposure was even higher among children aged 2 to 5, where 50% received at least one antibiotic prescription. Older patients showed even higher rates, with 47% of subjects over 65 years of age having received an antibiotic prescription, representing a 13.7% increase from the previous year [78].

The most frequently used antibiotic classes were penicillin combinations, including beta-lactams combinations (36%) and macrolides (25%), followed by third-generation cephalosporins (13.1%) and fluoroquinolones (10.5%). Conversely narrow-spectrum penicillins like amoxicillin were more rarely used, accounting for only 6.3% of prescriptions. These prescribing trends reflect a preference for broad-spectrum antibiotics over narrow-spectrum alternatives, with a ratio of 13.6, raising concerns about overuse and its potential contribution to antimicrobial resistance [78].

##### International Context

Inappropriate antibiotic prescribing is not unique to Italy, and similar trends have been observed in other countries. In France, acute viral RTIs have been identified as a major driver of antibiotic use in the outpatient setting, accounting for 17% of prescriptions in adults and 38% in children during the cold season [80]. In a Chinese study, antibiotics were prescribed in approximately 41% of outpatient visits for acute upper RTIs, highlighting the need for stewardship interventions [81]. Similarly, in the United States, a cohort study demonstrated widespread antibiotic overuse during influenza season among both pediatric and adult patients [82]. Collectively, these findings highlight the global nature of inappropriate antibiotic use and reinforce the importance of implementing robust antibiotic stewardship programs.

#### 3.3.2. Causes of Inappropriate Prescribing Patterns

The effectiveness of antibiotic therapy depends not only on selecting the appropriate agent but also on timely administration and dose optimization to maximize exposure while preserving the effectiveness of available treatments [83,84,85]. Inappropriate antibiotics use is often driven by insufficient knowledge of antibiotic classes and the pathogens responsible for specific infectious diseases, as well as poor understanding of dose optimization strategies. This knowledge gap can result in the prescription of inappropriate antibiotics for incorrect diagnoses (e.g., prescribing broad-spectrum antibiotics for viral infections), as well as errors in dosing and treatment duration [12,86,87,88].

While current guidelines provide valuable dosing recommendations, refinements may still be needed, especially since most antibiotics are developed primarily for adults, and the lack of pediatric-specific clinical trials already limits the availability of robust, evidence-based therapeutic options in this population [89]. As a result, pediatric dosing regimens are often extrapolated from adult data, leading to frequent off-label antibiotic prescriptions, modifications in dose, indication, or formulation. A thorough understanding of pharmacokinetic/pharmacodynamic (PK/PD) principles and the need for dose and duration adjustments in pediatric patients are therefore critical to achieve therapeutic targets while minimizing the risk of unnecessary toxicity [83].

#### 3.3.3. Optimizing Antibiotic Prescription: Practical Suggestions

To improve antibiotic prescribing patterns and help reduce antimicrobial resistance, experts in infectious diseases have developed two easy-to-use reference tables summarizing optimal antibiotic prescribing strategies across various clinical settings among children (Table 1) and adults (Table 2). These tables present experts’ insights based on literature and clinical experience, including first-line and alternative treatment options, choices for patients with allergies, and recommended treatment durations. Importantly, as these tables integrate both guideline-supported options and expert-derived suggestions, some regimens may differ from international guideline positions. These elements are noted within the tables and should be interpreted as expert suggestions. Specifically in Table 2 the q6 regimen for amoxicillin and amoxicillin clavulanate [72,73,74] and the q8 regimen for trimethoprim sulfamethoxazole [75,76,77] are suggested based on PK/PD evidence as highlighted in the table footnote.

Duration ranges refer to the infection and clinical scenario, not uniformly to all antibiotic classes. Specific agents may require different treatment durations according to PK/PD characteristics and guideline recommendations.

#### 3.3.4. Insight from Adult Patients: PK/PD Optimization

As previously mentioned, understanding key PK/PD principles is critical to achieving therapeutic targets while minimizing the risk of unnecessary toxicity. In this context, the optimization of beta-lactam antibiotics dosing warrants special consideration due to their time-dependent activity, meaning their efficacy depends on how long plasma concentrations remain above the pathogen’s minimum inhibitory concentration (MIC). For this reason, and based on their clinical experience, experts highlight that fractionated dosing is the preferred strategy for beta-lactams, as it helps maintain effective drug levels. For example, amoxicillin and amoxicillin/clavulanate, when administered every six–eight hours, more effectively sustain plasma concentrations above the MIC. This is especially important for pathogens with reduced susceptibility, which have elevated MIC values compared to wild-type strains, as well as for deep-seated or biofilm-associated infections, such as otitis media, sinusitis, and tonsillar abscesses [90].

A crucial concern is that if antibiotic concentrations fall within the mutant selection window (MSW)—the range between the MIC and the mutant prevention concentration (MPC)—it creates favorable conditions for selecting resistant bacterial strains [91,92]. This issue is further emphasized by the European Committee on Antimicrobial Susceptibility Testing (EUCAST) revision of MIC values, particularly in light of “MIC creep,” a phenomenon defined as a gradual and unnoticed increase in MIC values due to prolonged antibiotic pressure [93]. Recognizing these concerns, international guidelines, including those from the Infectious Diseases Society of America (IDSA)/American Thoracic Society (ATS), and the NICE, already recommend fractionated dosing in adults to optimize therapeutic outcomes and minimize resistance [94,95]. According to the experts, applying this strategy to pediatric care may also be beneficial.

Oral cephalosporins present additional challenges due to their poor bioavailability, which can limit their effectiveness in pediatric infections. When oral cephalosporins are necessary, those with the highest bioavailability should be prioritized to ensure optimal drug exposure and clinical efficacy. Table 3 provides an overview of the oral bioavailability for commonly used oral cephalosporins, serving as a reference for appropriate antibiotic selection in pediatric care (Table 3) [96,97,98,99,100,101,102].

#### 3.3.5. Insight from Adult Patients: Treatment Duration

Another essential aspect in antibiotic therapy optimization is treatment duration. In the opinion of the experts, a major limitation of many guidelines is their tendency to recommend a fixed duration, despite literature evidence supporting a more individualized approach, tailored to each patient’s condition. Recent studies in adult patients suggest the possibility to shift toward shorter treatment durations across various infections, as these regimens show comparable efficacy and fewer adverse effects compared with longer courses [103,104,105,106].

In adult patients with RTIs, multiple systematic reviews suggest that shorter treatment durations (5–7 days) are as effective as longer courses in the outpatient setting, with an improved safety profile and reduced treatment burden in adult patients [103,104,105]. Similarly, a retrospective study on outpatient antibiotic prescriptions in older Canadian adults with pneumonia supports 3- to 5-day treatment courses as a clear target for stewardship efforts [106]. The IDSA also recommends shorter treatment durations (5–7 days) for acute bacterial rhinosinusitis in adults, citing similar efficacy and fewer side effects compared to prolonged therapy [107].

Experts further highlight the importance of appropriate dosing and duration as key targets of antibiotic stewardship programs for UTIs. Different studies have reported that shorter treatment courses (approximately 3 days) as are effective as longer ones (more than 5–7 days) in the treatment of uncomplicated cystitis in women [108]. Shorter courses may also be a viable option for UTIs in men, although data remain limited and the optimal duration has yet to be established [108].

Although most studies on shorter duration focus on adult populations, some evidence is also emerging for children. For instance, a systematic review on the use of antibiotics in pediatric patients with otitis media suggests that in certain subgroups (children > 2 years with mild symptoms), shorter regimens (5–7 days) are effective, reducing overall antibiotic exposure [109]. A short antibiotic course (3 to 5 days) has also been validated by multiple studies and metanalyses for the treatment of non-complicated pneumonia in children for whom an accurate follow-up within 72 h can be granted [110], and this indication has been recently included in a first clinical practice guidelines in Italy [111].

## 4. Study Limitations

This review has several limitations. It is based on a narrative, evidence-informed approach and does not follow a formal systematic methodology. Furthermore, it places greater emphasis on pediatric populations, partly reflecting the greater availability of literature in this group, whereas evidence on the use of anti-inflammatory drugs and paracetamol in adult patients is comparatively less represented. Moreover, although it draws on an international body of literature, it primarily focuses on the Italian clinical context—particularly with regard to antibiotic prescribing patterns—reflecting the clinical background of the contributing authors. These factors may limit the generalizability of some findings to other countries and healthcare settings.

## 5. Conclusions

The rational use of antipyretics and anti-inflammatory drugs is essential for managing fever and pain in infectious diseases, particularly in children. Based on literature evidence on the potential safety risks associated with ibuprofen, experts suggest that paracetamol should be the preferred first-line agent due to its favorable safety profile, while ibuprofen should be used with caution, especially during active infections or in patients at risk of renal or gastrointestinal complications. In line with current guidelines, experts also emphasize that inappropriate use, such as alternating or combining antipyretics or their prophylactic use around vaccination, should be avoided, as these practices do not improve clinical outcomes and may increase the risk of adverse effects.

Equally important is the rational use of antibiotics, which should be prescribed only when clinically indicated, with careful selection of the appropriate agent to optimize outcomes and minimize resistance. Expert insights suggest that antibiotic stewardship programs should incorporate strategies such as fractionated dosing, consideration of drug bioavailability, and, where appropriate, shorter treatment durations to maintain efficacy and reduce toxicity.

This paper offers expert-informed, evidence-based insights to support appropriate prescribing in adults and children with infectious diseases, with the aim of improving clinical outcomes, and contributing to the global effort against antimicrobial resistance.

## Figures and Tables

**Table 1:** Antibiotic and antipyretic use in Pediatric Infections.

	Antibiotics				Antipyretics
Pathology	First-Line Treatment	Second-Line Treatment	If Allergic	Duration of First-Line Treatment	
Acute Otitis Media (Uncomplicated, No Risk Factors, Mild Symptoms) Empiric therapy	Amoxicillin 75–90 mg/kg/day in 3 doses or Amoxicillin/Clavulanate 90 mg/kg/day in 3 doses if not adequately vaccinated for Haemophilus influenzae	Amoxicillin/Clavulanate 90 mg/kg/day in 3 doses	Non-IgE-mediated Penicillin Allergy: Cefuroxime 30 mg/kg/day in 2 doses (max 500 mg/day) IgE-mediated Penicillin Allergy or Unknown: Azithromycin 10 mg/kg/day or Clarithromycin 15 mg/kg/day in 2 doses (max 1 g/day)	5 days if no risk (>2 years, no otorrhea, unilateral) 7–10 days if high-risk progres- sion (<2 years or otorrhea)	For all infections, in case of fever > 38 °C or discomfort, administer paracetamol 10 mg/kg if weight < 5 kg or 15 mg/kg if weight > 5 kg
Acute Otitis Media (Perforated Membrane, Recent Antibiotic The- rapy, Severe Symptoms, Recurrences, First-line Ineffective)	Amoxicillin/Clavulanate 75–90 mg/kg/day in 3 doses	Ceftriaxone 50 mg/kg/day IV or IM (max 1 g/day)	Non-IgE-mediated Penicillin Allergy: Cefuroxime 30 mg/kg/day in 2 doses (max 500 mg/day) IgE-mediated Penicillin Allergy or Unknown: Azithromycin 10 mg/kg/day on day 1 (max 500 mg/day), then 5 mg/kg/day from day 2–5 in 1 dose or Clarithromycin 15 mg/kg/day in 2 doses (max 1 g/day) or Fluoroquinolone	10 days	
Pharyngitis/Tonsillitis (*S. pyogenes*)	Amoxicillin 50 mg/kg/day in 2–3 doses	-	First-generation cephalosporins. Macrolides (Clarithromycin 15 mg/kg/day in 2 doses or Azithromycin 10 mg/kg/day every 24 h) only if IgE-mediated allergy proven	10 days (5–7 days in settings with low rheumatic fever prevalence and according to specific and local GLs)	
Community-Acquired Pneumonia (<5 years)	Amoxicillin 90 mg/kg/day in 3 doses	-	Second- or third-generation cephalosporins. Macrolides (Clarithromycin 15 mg/kg/day in 2 doses or Azithromycin 10 mg/kg/day every 24 h) only if IgE-mediated allergy proven	5 days	
Community-Acquired Pneumonia (>5 years)	Amoxicillin 90 mg/kg/day in 3 doses +/− Macrolides (Clarithromycin 15 mg/kg/ day in 2 doses or Azithromycin 10 mg/kg/day every 24 h) if no response after 48–72 h	-	-	7–10 days	
Pneumonia (Streptococcus pneumoniae)	Amoxicillin 90 mg/kg/day in 3 doses	Ceftriaxone 75–100 mg/kg/day or Cefotaxime 150 mg/kg/day in 3 doses if hospitalized	Second- or third-generation cephalosporins Macrolides (Clarithromycin 15 mg/kg/day in 2 doses or Azithromycin 10 mg/kg/day every 24 h) only if IgE-mediated allergy proven Levofloxacin or Clindamycin as alternatives	5 days	
Pneumonia (Mycoplasma pneumoniae)	Clarithromycin 15 mg/kg/day in 2 doses or Azithromycin 10 mg/kg/day every 24 h	-	-	5 days	
Febrile UTI (<1 month old) *	Ampicillin 50 mg/kg/day IV every 6–8 h + Gentamicin 4 mg/kg/day IV	Ampicillin 50 mg/kg/day IV every 6–8 h + Netilmicin 4 mg/kg/day	-	3–4 days IV, then switch to oral therapy (Total: 10–14 days)	
Febrile UTI (1–3 months old) *	Cefotaxime 150–200 mg/kg/day in 3 doses or Ceftriaxone 75–100 mg/kg/day IV	-	-	If afebrile for 24 h, switch to oral therapy based on antibiogram	
Febrile UTI (>3 months old, well-appearing)	Amoxicillin/Clavulanate 50–90 mg/kg/day in 3 doses PO	Cefixime 4 mg/kg/dose every 12 h or Ceftibuten 9 mg/kg/dose every 12 h	-	10 days	
Febrile UTI (>3 months old, ill-appearing)	Amoxicillin/Clavulanate 100 mg/kg/day in 3 doses IV (or Ampicillin/Sulbactam)	Cefotaxime 150–200 mg/kg/day in 3 doses or Ceftriaxone 75–100 mg/kg/day IV	-	If afebrile for 24 h, switch to oral therapy based on antibiogram. If febrile 10 days, if urosepsis 14 days.	

GLs: guidelines; Ig: immunoglobulin; IM: intramuscular; IV: intravenous; PO: Per Os; UTI: urinary tract infection. * Ibuprofen is contraindicated in infants < 3 months.

**Table 2:** Antibiotic and antipyretic use in Adult Infections.

	Antibiotics				Antipyretics
Pathology	First-Line Treatment	Second-Line Treatment	If Allergic	Duration of First-Line Treatment	
Acute Otitis Media (Uncomplicated, No Risk Factors, Mild Symptoms) Empiric therapy	No Atb-tx (48–72 h) If not symptoms remission (48–72 h) Amoxicillin 1000 mg q6–8h *	If not symptoms remission (48–72 h) Amoxicillin/Clavulanate 875/125 mg q6–8h *	Penicillin Allergy: Clarithromycin 500 mg q12h or Azithromycin 500 mg qd	5–7 days	For all infections, in case of fever > 38 °C or discomfort, administer paracetamol 500–1000 mg max 3 g/day
Acute Otitis Media Complicated (Perforated Membrane, Severe Symptoms, Recurrences)	Amoxicillin/Clavulanate 875/125 mg q6h *	Cefditoren pivoxil 400 mg q12h Or Ceftriaxone 2 g/day IV or IM	Penicillin Allergy: Clarithromycin 500 mg q12h or Azithromycin 500 mg qd Or Doxycycline 100 mg q12h	7–10 days	
Pharyngitis/Tonsillitis (*S. pyogenes*)	Amoxicillin 1000 mg q6–8h *	-	Clarithromycin 500 mg q12h Azithromycin 500 mg qd	3–5 days (stop therapy 48 h after symptom remission)	
Community-Acquired Pneumonia	Amoxicillin/Clavulanate 875/125 mg q6h * +/− Clarithromycin 500 mg q12h or Azithromycin 500 mg qd	Ceftriaxone 2 g qd +/− Clarithromycin 500 mg q12h or Azithromycin 500 mg qd	Clarithromycin 500 mg q12h Azithromycin 500 mg qd Or Doxycycline 100 mg q12h Or Moxifloxacine 400 mg qd	3–5 days (stop therapy 48 h after symptom remission)	
Atypical Pneumonia (Mycoplasma pneumoniae or Chlamydia)	Clarithromycin 500 mg q12h or Azithromycin 500 mg qd			5 days	
UTI uncomplicated	Nitrofurantoin 50–100 mg q6h Or TMP-SMX 160/800 mg 1 cp q12h	Fosfomycin tromethamol 3 g qd		3 days (1 day fosfomycin tromethamol if young female with episodic cystitis)	
Pyelonephritis	Amoxicillin/Clavulanate 875/125 mg q6h *	Levofloxacin 500 mg q12h or Ciprofloxacin 750–500 mg q12h	3° line TMP-SMX 800/160 mg 1 cp q8h **	10–14 days	
Prostatitis	Levofloxacin 500 mg q12h or Ciprofloxacin 750–500 mg q12h	TMP-SMX 800/160 mg 1 cp q8h **	3° line Doxycycline 100 mg q12h	21–28 days	

Atb-tx: antibiotic therapy; IM: intramuscular; IV: intravenous; TMP-SMX: Trimethoprim–Sulfamethoxazole; UTI: urinary tract infection. Asterisks indicate expert-derived dosing suggestions based on literature evidence and clinical experience, which may diverge from international guideline recommendations. * For amoxicillin and amoxicillin/clavulanate, the q6 dosing regimen is not reported in the summary of product characteristics but is supported by PK/PD evidence [72,73,74]. ** For trimethoprim/sulfamethoxazole, the q8 dosing regimen is not reported in the summary of product characteristics but is supported by PK/PD evidence [75,76,77].

**Table 3:** Oral bioavailability of most commonly used cephalosporins.

Cephalosporin	Oral Bioavailability (Approximate)	Notes
Cefuroxime axetil [96]	30–50%	Bioavailability is variable and improves with food (up to ~50% under optimal conditions).
Cefixime [97]	40–50%	Median values reported; values can vary depending on formulation and intake conditions.
Cefpodoxime proxetil [98]	~50%	Absorption improves with food; variability observed based on nutritional status.
Ceftibuten [99]	70–90%	Among the best-absorbed oral cephalosporins; high bioavailability.
Cefdinir [99]	16–21%	Lower bioavailability compared to other oral cephalosporins; may be influenced by interaction with food or other factors.
Cefaclor [100]	60–75%	Good bioavailability, values can vary depending on formulation and intake conditions.
Cefadroxil [101,102]	~90%	High bioavailability; suitable for pediatric use.

Note: Average values are reported, this may vary based on several factors (e.g., nutritional status, specific formulation, intake conditions, age, gastrointestinal function, etc.).

## Data Availability

No new data were created or analyzed in this study. Data sharing is not applicable to this article.
